# Supplementary material for: Comprehensive Chloroplast Genomic Insights into Amaranthus: Resolving the Phylogenetic and Taxonomic Status of A. powellii and A. bouchonii
Source: Plants (Basel). 2025 Feb 20;14(5):649. doi: 10.3390/plants14050649 (PMC11902225; doi:10.3390/plants14050649)
Supplement: Supplementary file 1 [file plants-14-00649-s001.zip › plants-3344668-supplementary/Figure 1.pdf]

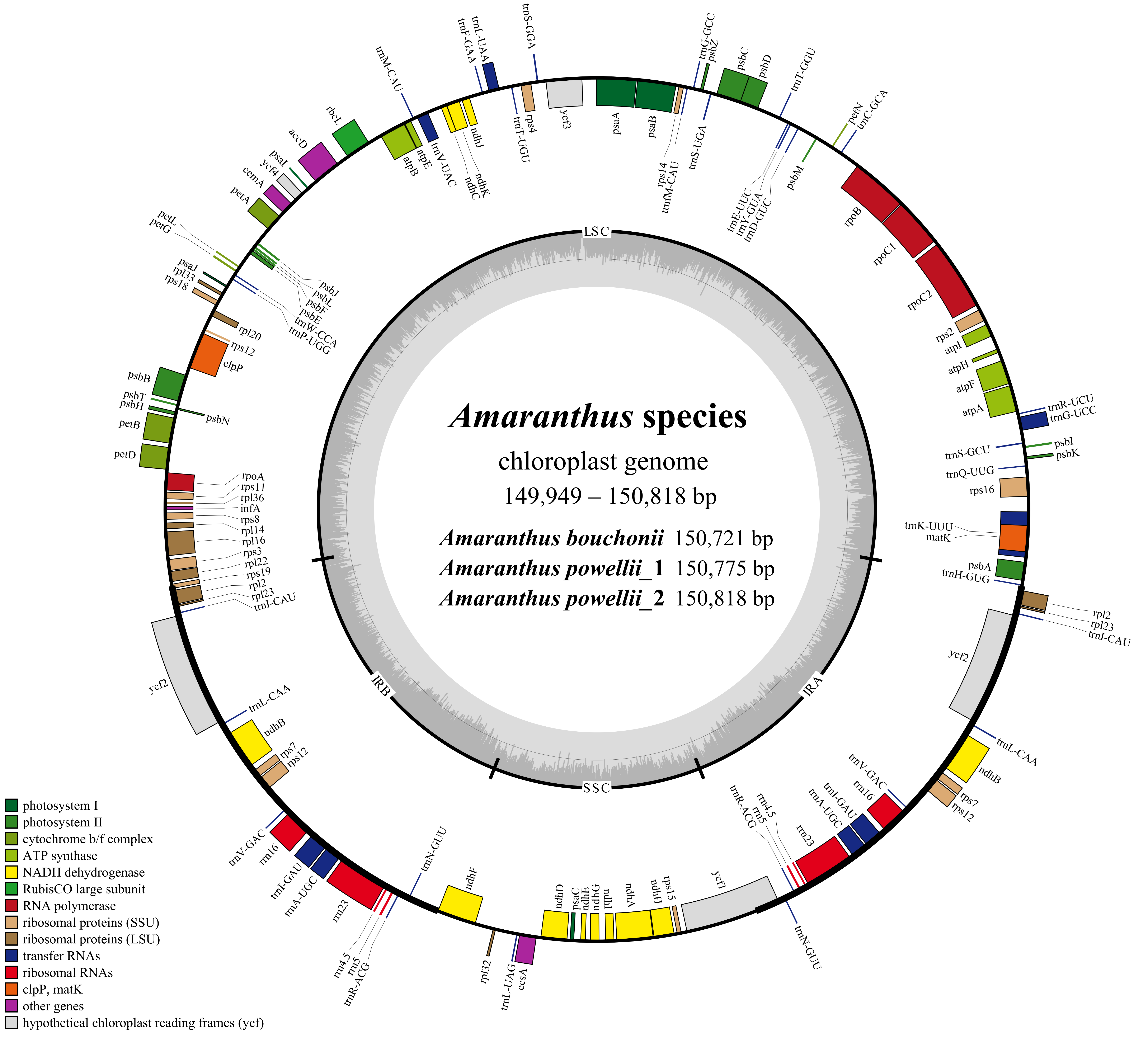
Amaranthus species
chloroplast genome
149,949 – 150,818 bp
Amaranthus bouchonii 150,721 bp
Amaranthus powellii_1 150,775 bp
Amaranthus powellii_2 150,818 bp
LSC
SSC
IRA
IRB
photosystem I
photosystem II
cytochrome b/f complex
ATP synthase
NADH dehydrogenase
RubisCO large subunit
RNA polymerase
ribosomal proteins (SSU)
ribosomal proteins (LSU)
transfer RNAs
ribosomal RNAs
clpP, matK
other genes
hypothetical chloroplast reading frames (ycf)
trnM-CAU
trnF-GAA
trnL-UAA
trnS-GGA
trnT-UGU
rps4
ycf3
psaA
psaB
rps14
trnfM-CAU
trnG-GCC
psbZ
trnS-UGA
psbC
psbD
trnT-GGU
trnE-UUC
trnY-GUA
trnD-GUC
psbM
petN
trnC-GCA
rpoB
rpoC1
rpoC2
rps2
atpI
atpH
atpF
atpA
trnR-UCU
trnG-UCC
trnS-GCU
psbI
psbK
trnQ-UUG
rps16
trnK-UUU
matK
psbA
trnH-GUG
rpl2
rpl23
trnI-CAU
ycf2
trnL-CAA
ndhB
rps7
rps12
trnV-GAC
rrn16
trnI-GAU
trnA-UGC
rrn23
rrn4.5
rrn5
trnR-ACG
trnN-GUU
ycf1
rps15
ndhH
ndhA
ndhI
ndhG
ndhE
psaC
ndhD
ccsA
trnL-UAG
rpl32
ndhF
rbcL
accD
psaI
ycf4
cemA
petA
petL
petG
psbJ
psbL
psbF
psbE
trnW-CCA
trnP-UGG
psaJ
rpl33
rps18
rpl20
clpP
psbB
psbT
psbH
psbN
petB
petD
rpoA
rps11
rpl36
infA
rps8
rpl14
rpl16
rps3
rpl22
rps19
atpB
atpE
trnV-UAC
ndhC
ndhK
ndhJ
