# Supplementary figures and images for: Comprehensive Chloroplast Genomic Insights into Amaranthus: Resolving the Phylogenetic and Taxonomic Status of A. powellii and A. bouchonii
Source: Plants (Basel). 2025 Feb 20;14(5):649. doi: 10.3390/plants14050649 (PMC11902225; doi:10.3390/plants14050649)

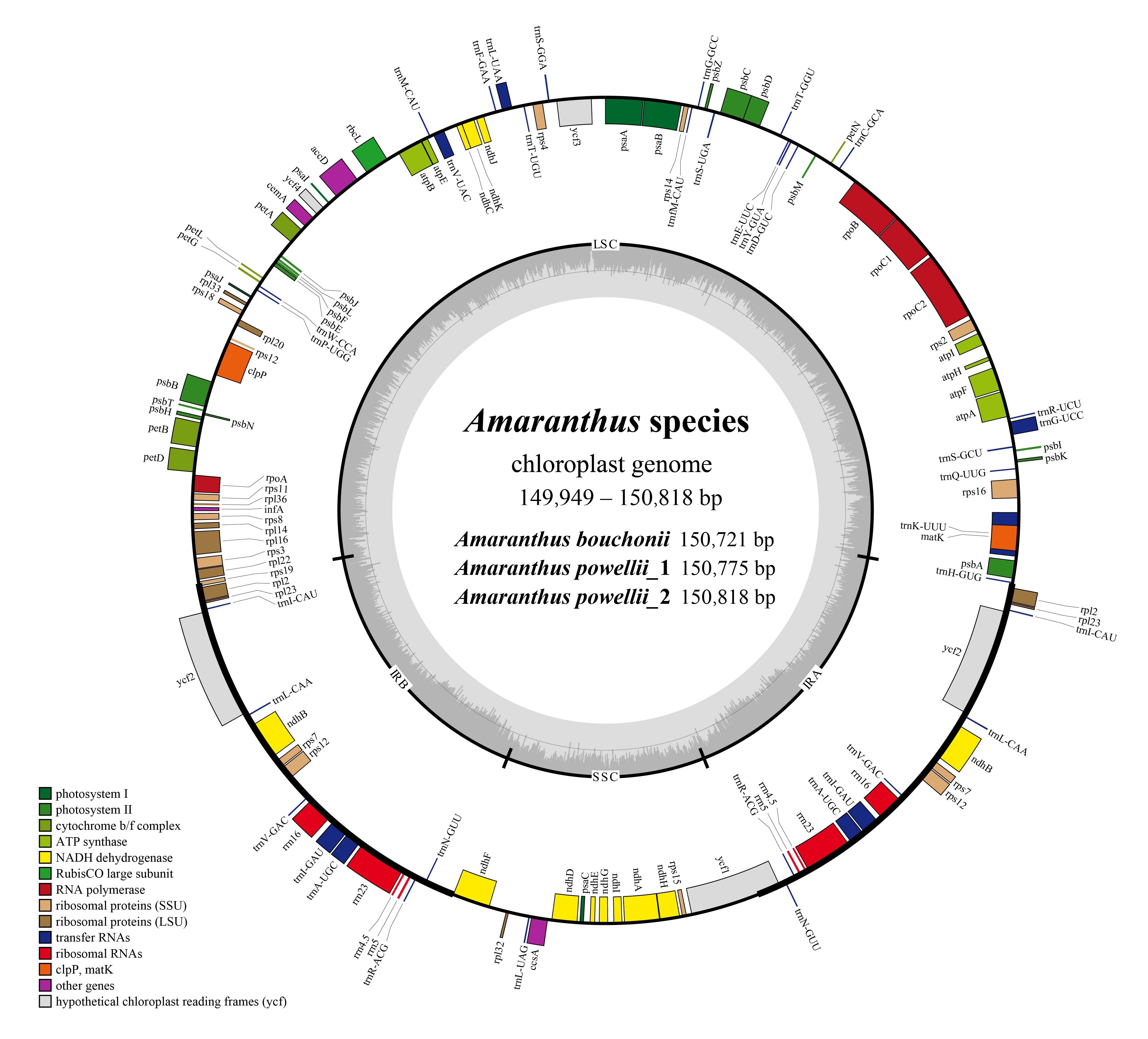

Supplement: Supplementary file 1 [file plants-14-00649-s001.zip › plants-3344668-supplementary/Figure 1.jpg]

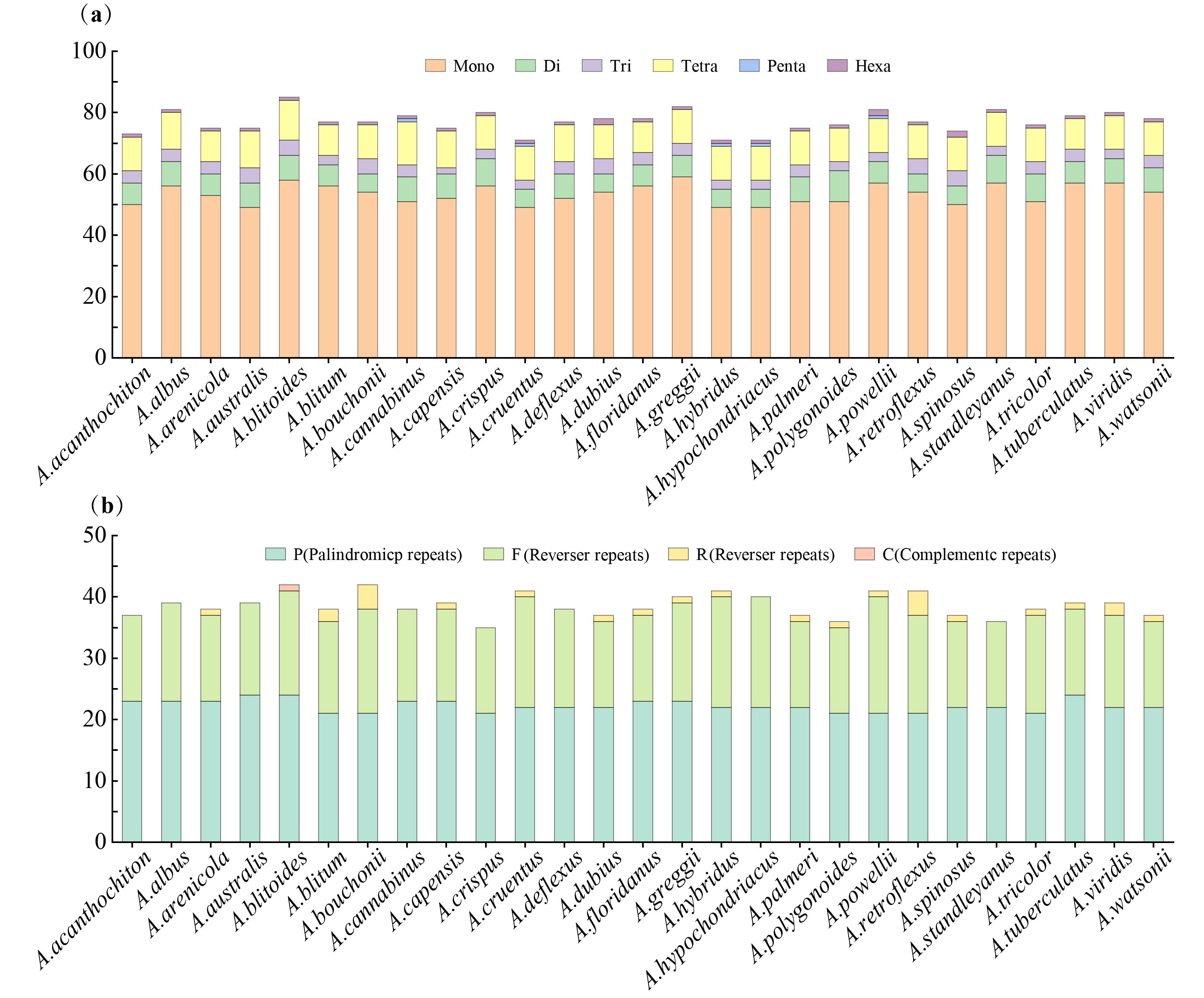

Supplement: Supplementary file 1 [file plants-14-00649-s001.zip › plants-3344668-supplementary/Figure 2.jpg]

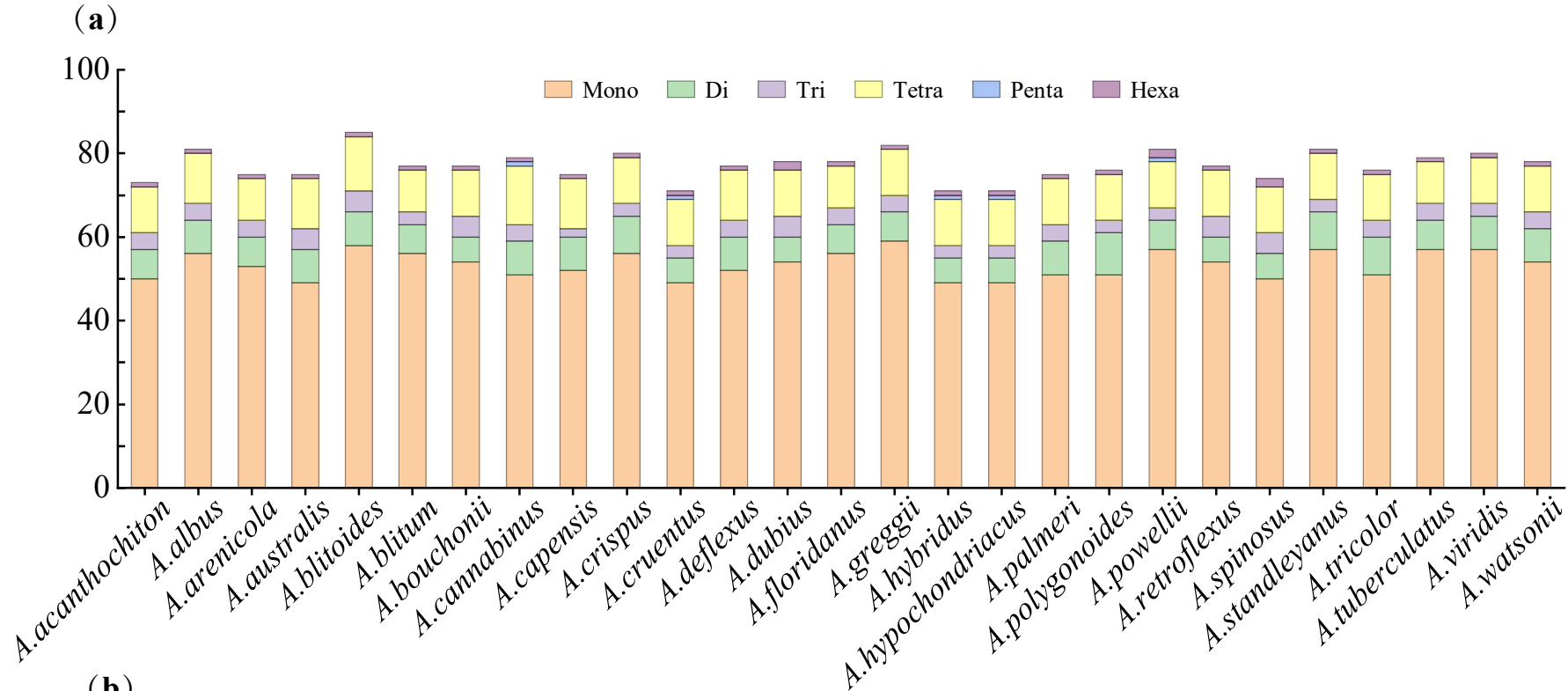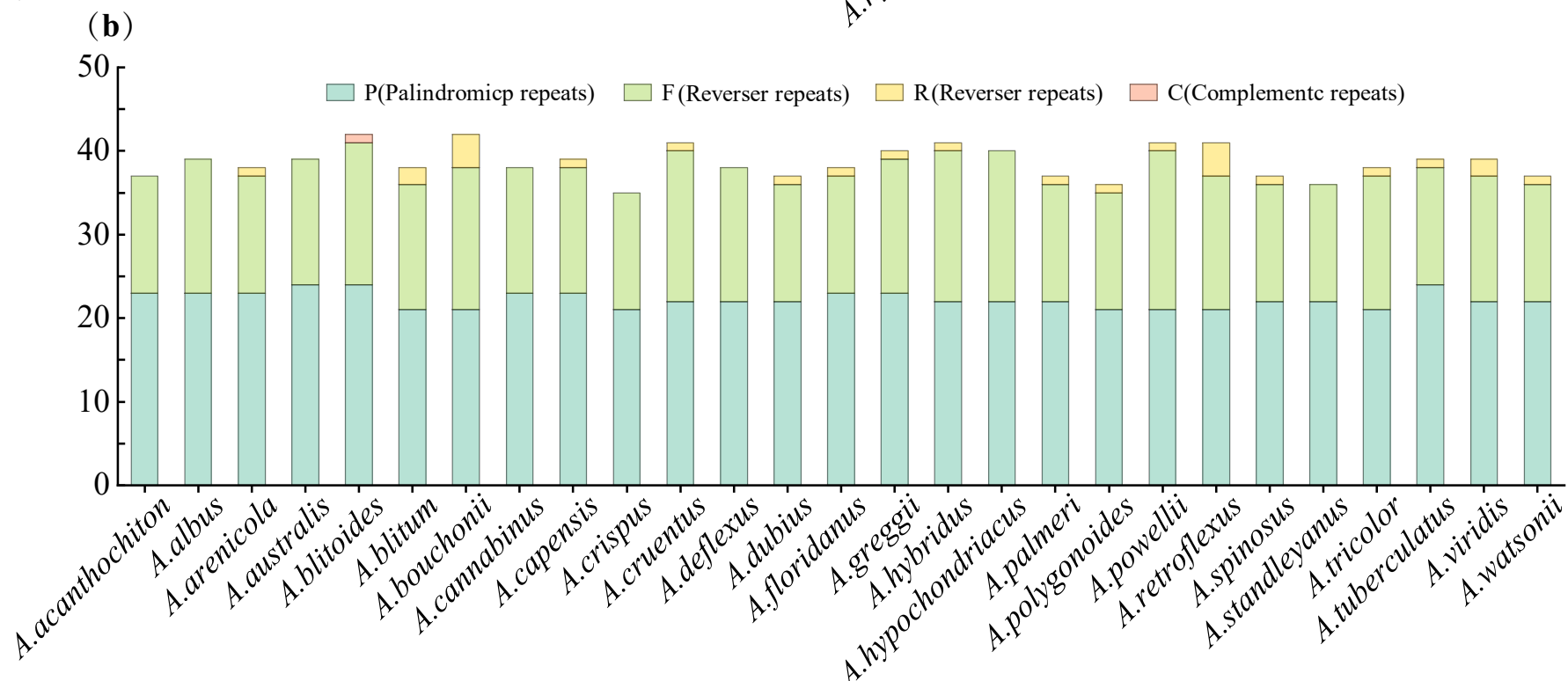

Supplement: Supplementary file 1 [file plants-14-00649-s001.zip › plants-3344668-supplementary/Figure 2.pdf]

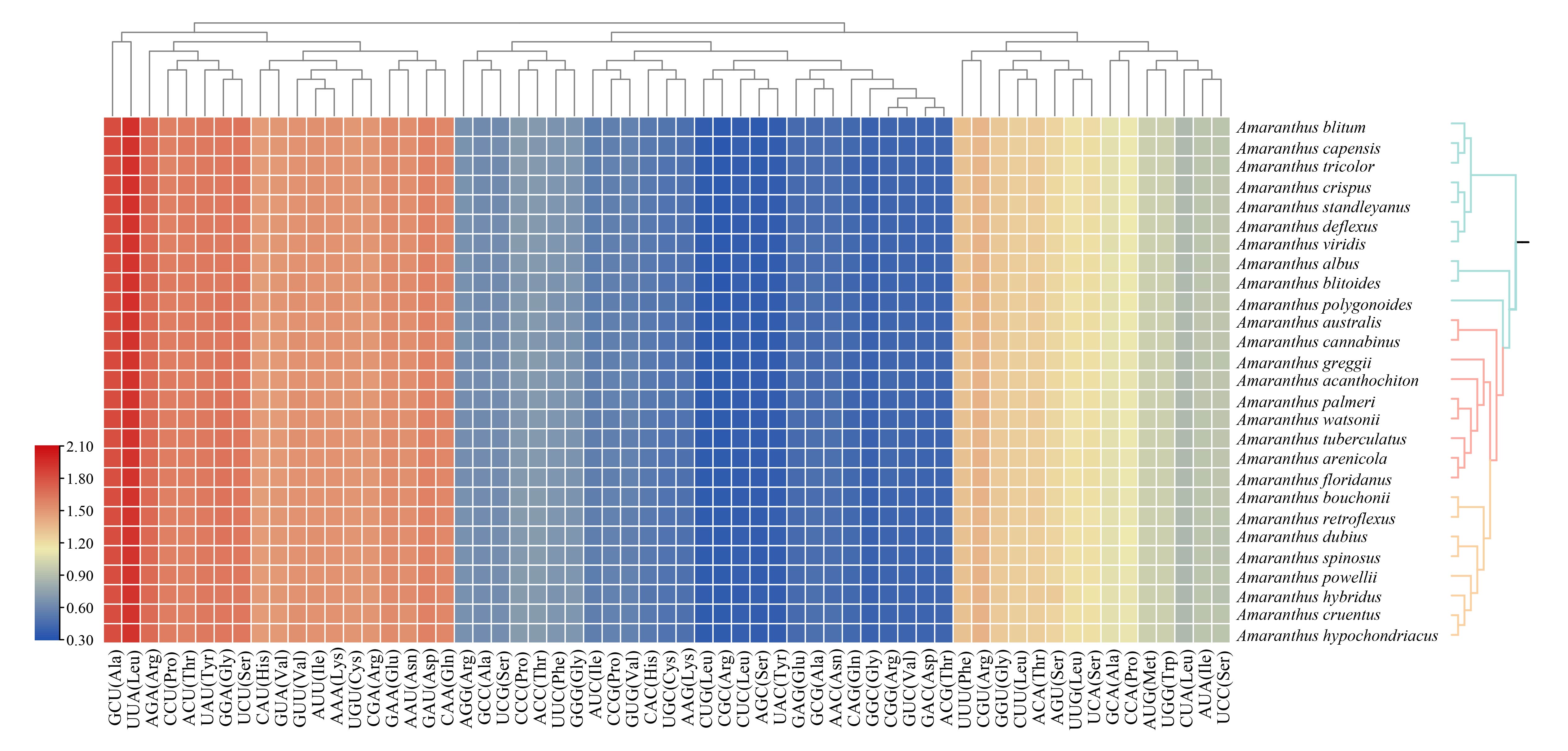

Supplement: Supplementary file 1 [file plants-14-00649-s001.zip › plants-3344668-supplementary/Figure 3.jpg]

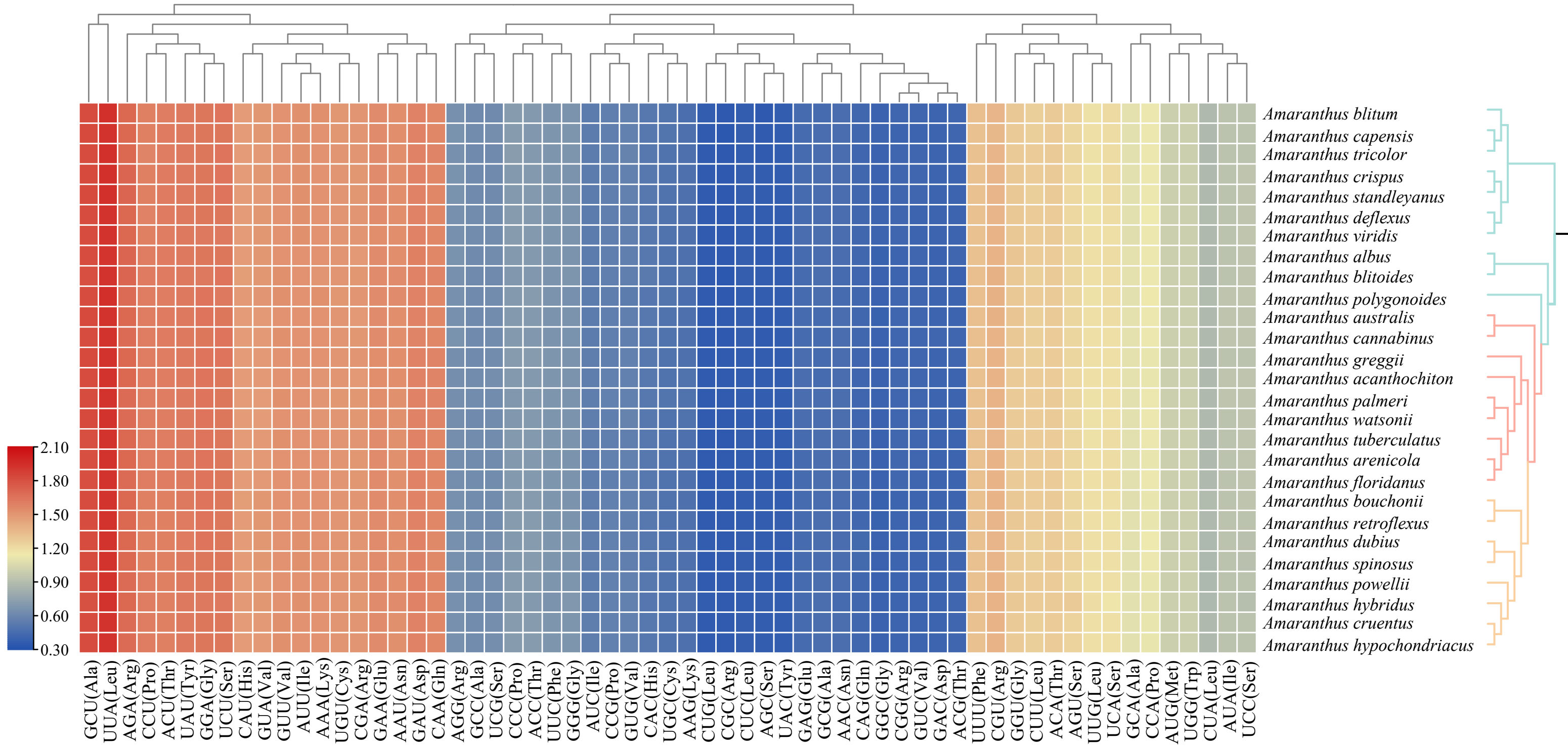

Supplement: Supplementary file 1 [file plants-14-00649-s001.zip › plants-3344668-supplementary/Figure 3.pdf]

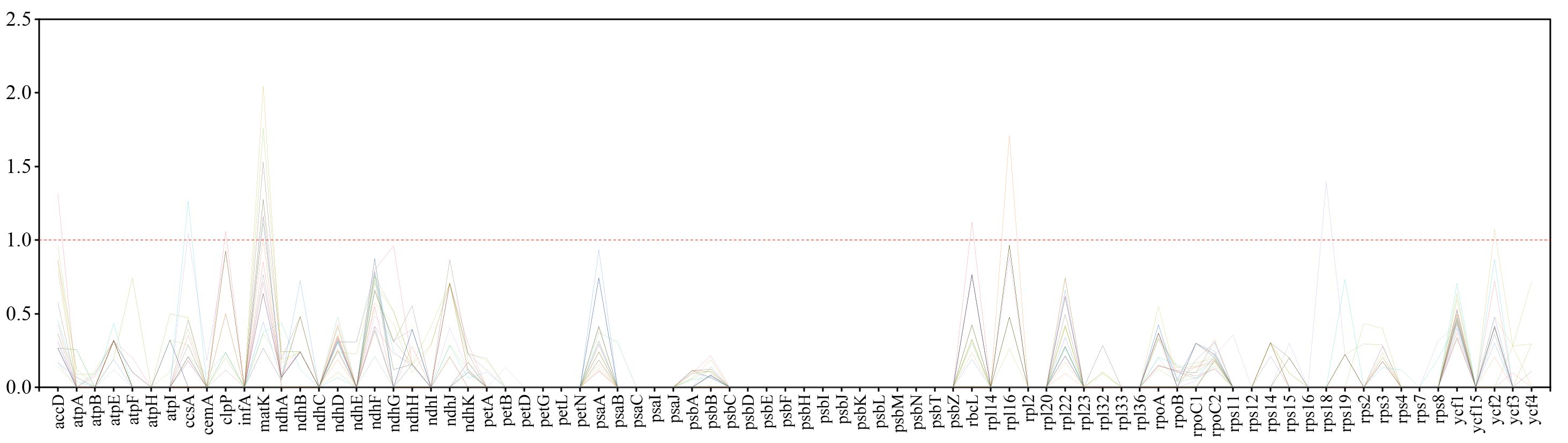

Supplement: Supplementary file 1 [file plants-14-00649-s001.zip › plants-3344668-supplementary/Figure 4.jpg]

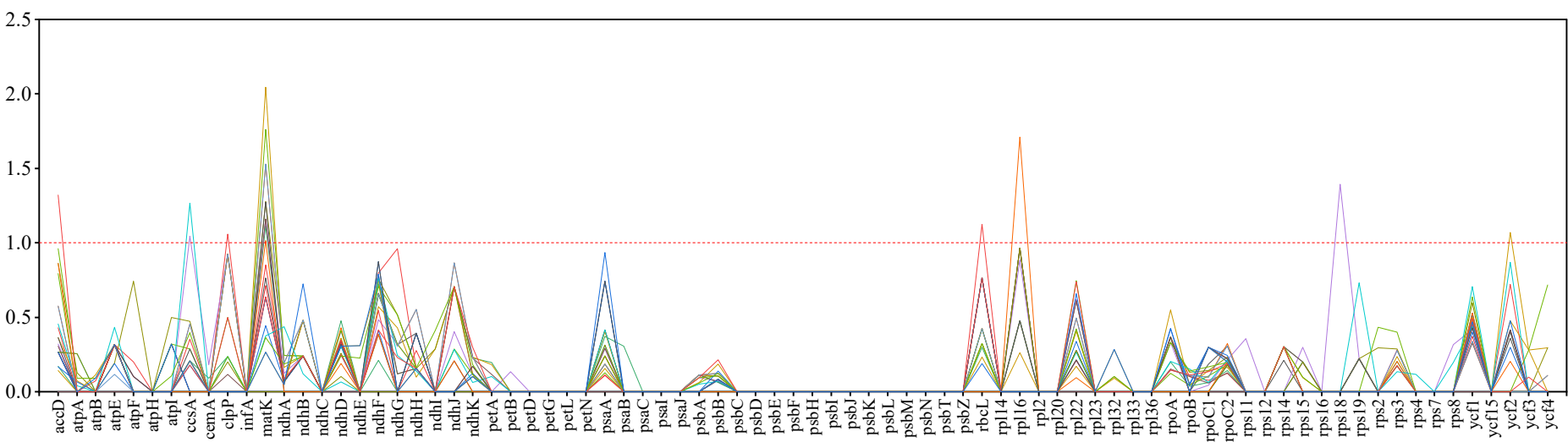

Supplement: Supplementary file 1 [file plants-14-00649-s001.zip › plants-3344668-supplementary/Figure 4.pdf]

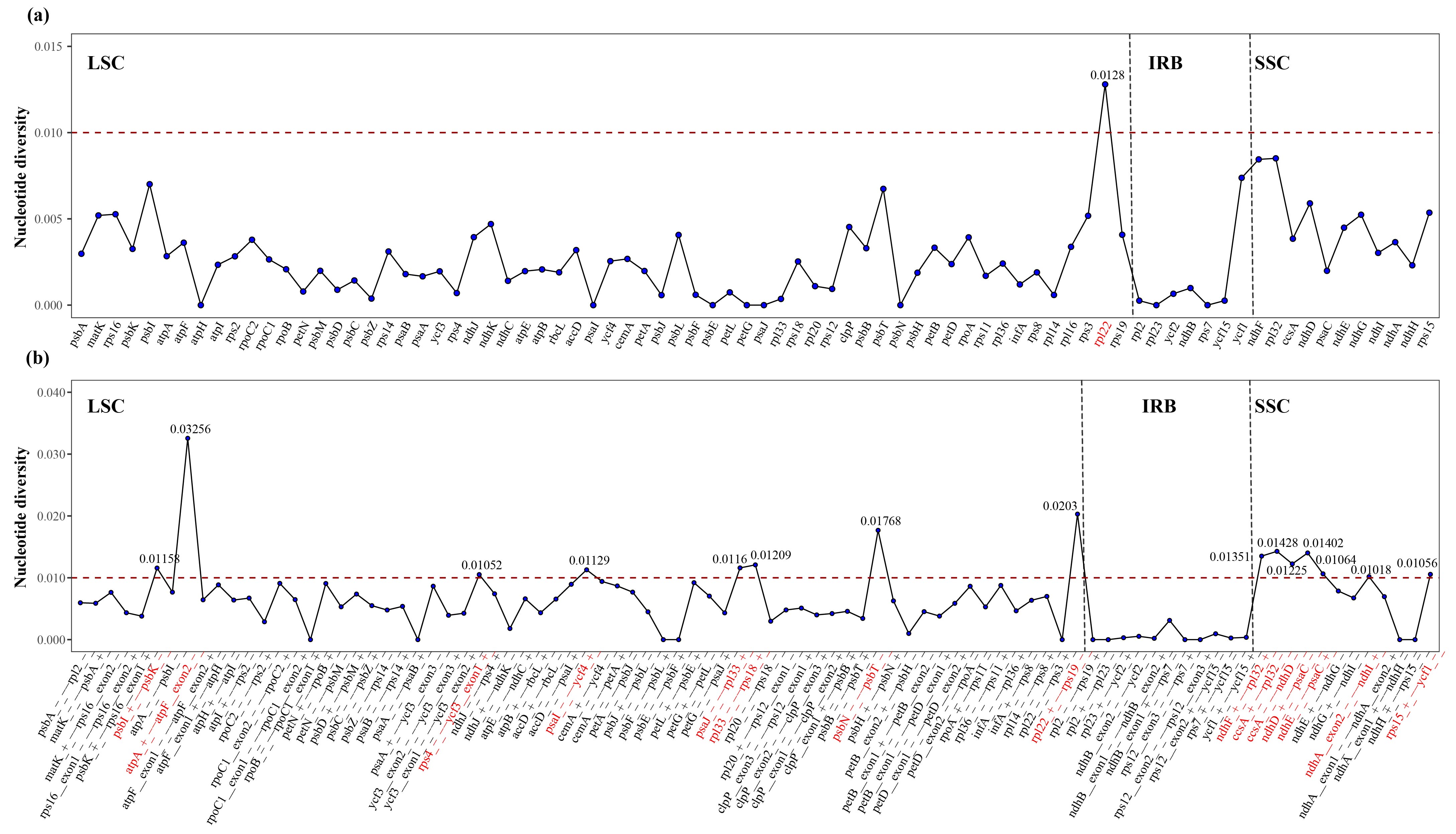

Supplement: Supplementary file 1 [file plants-14-00649-s001.zip › plants-3344668-supplementary/Figure 5.jpg]

**(a)**

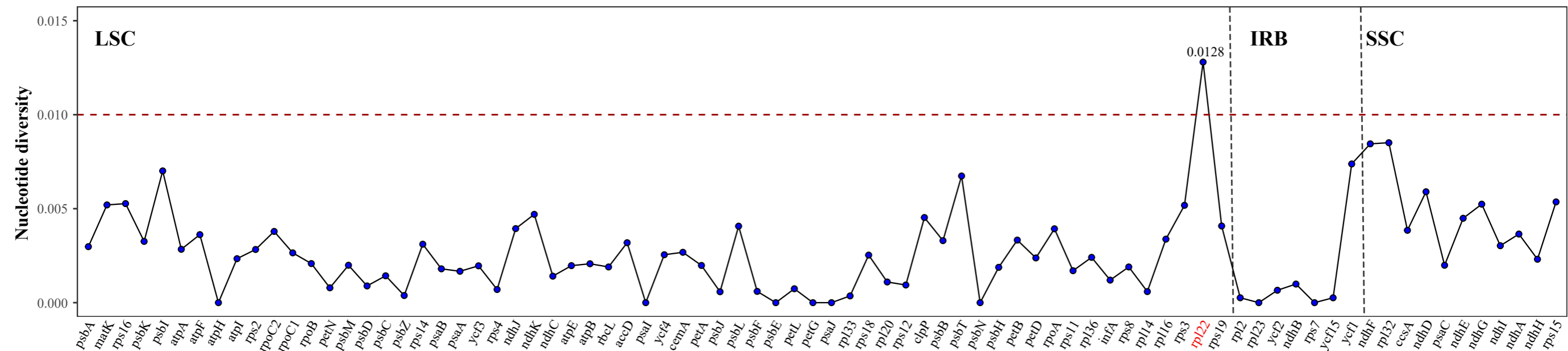

(b)

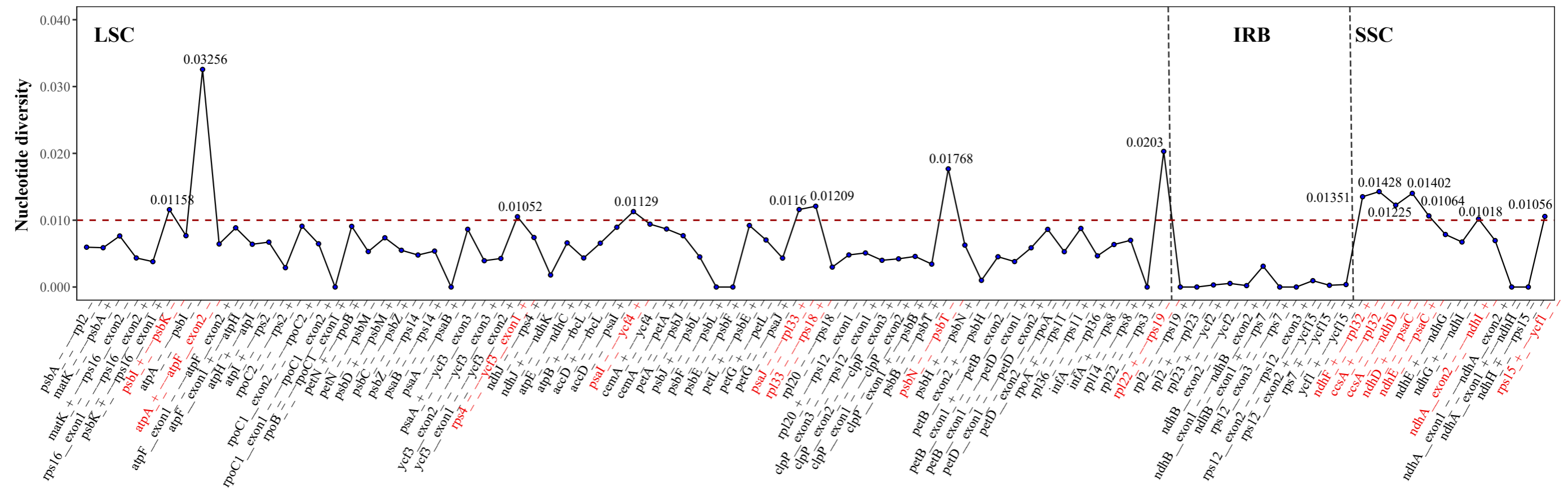

Supplement: Supplementary file 1 [file plants-14-00649-s001.zip › plants-3344668-supplementary/Figure 5.pdf]

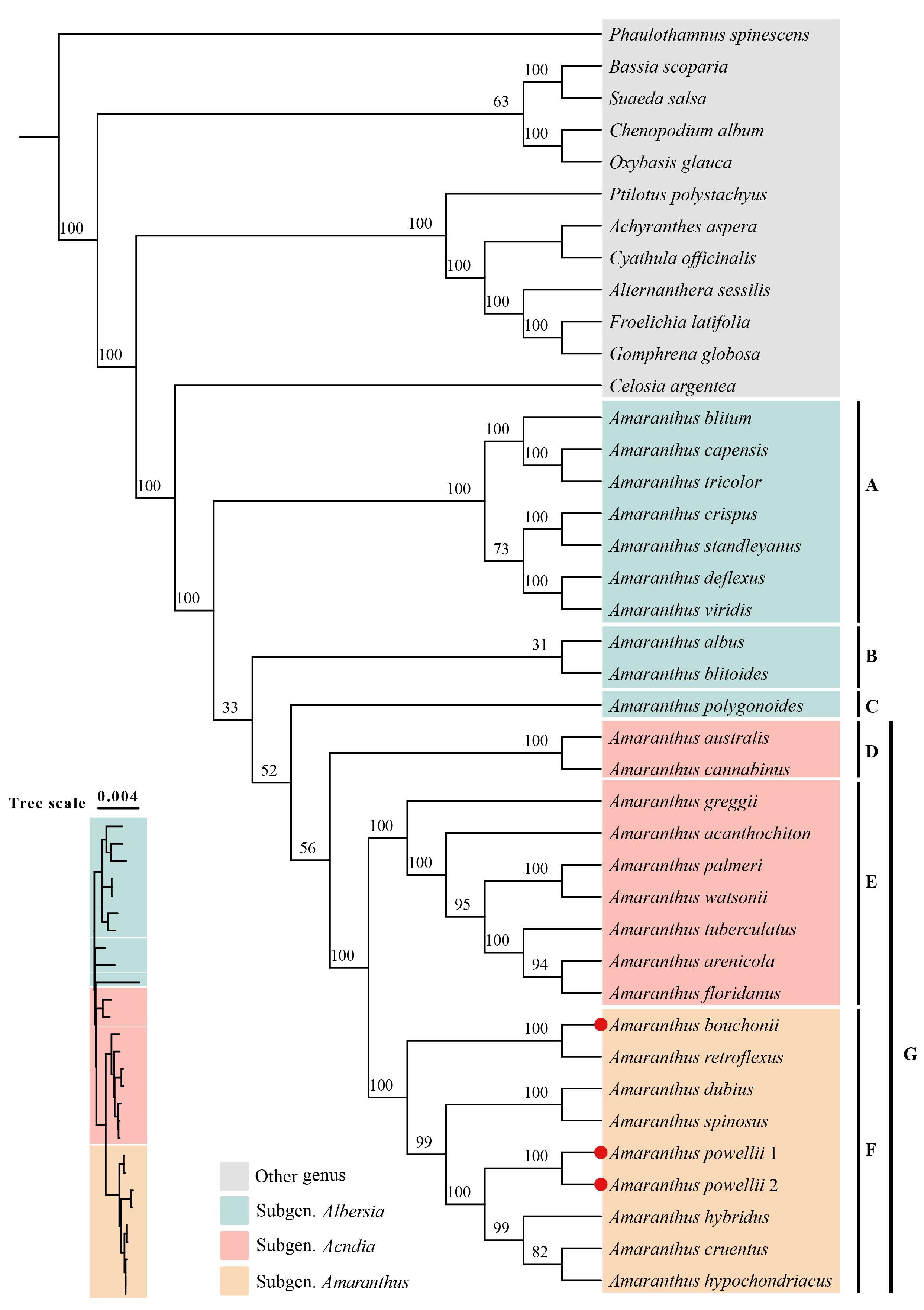

Supplement: Supplementary file 1 [file plants-14-00649-s001.zip › plants-3344668-supplementary/Figure 6.jpg]

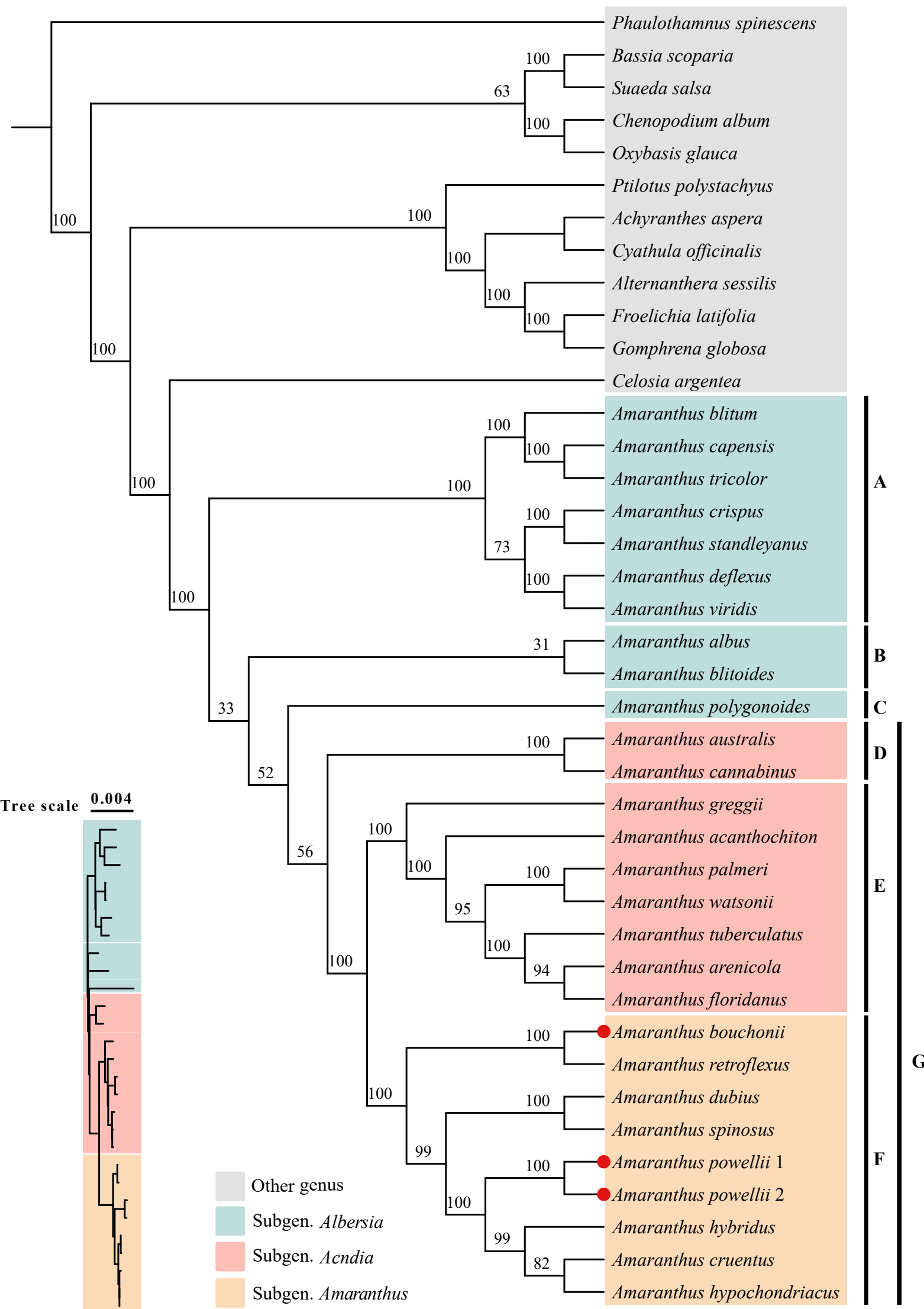

Supplement: Supplementary file 1 [file plants-14-00649-s001.zip › plants-3344668-supplementary/Figure 6.pdf]

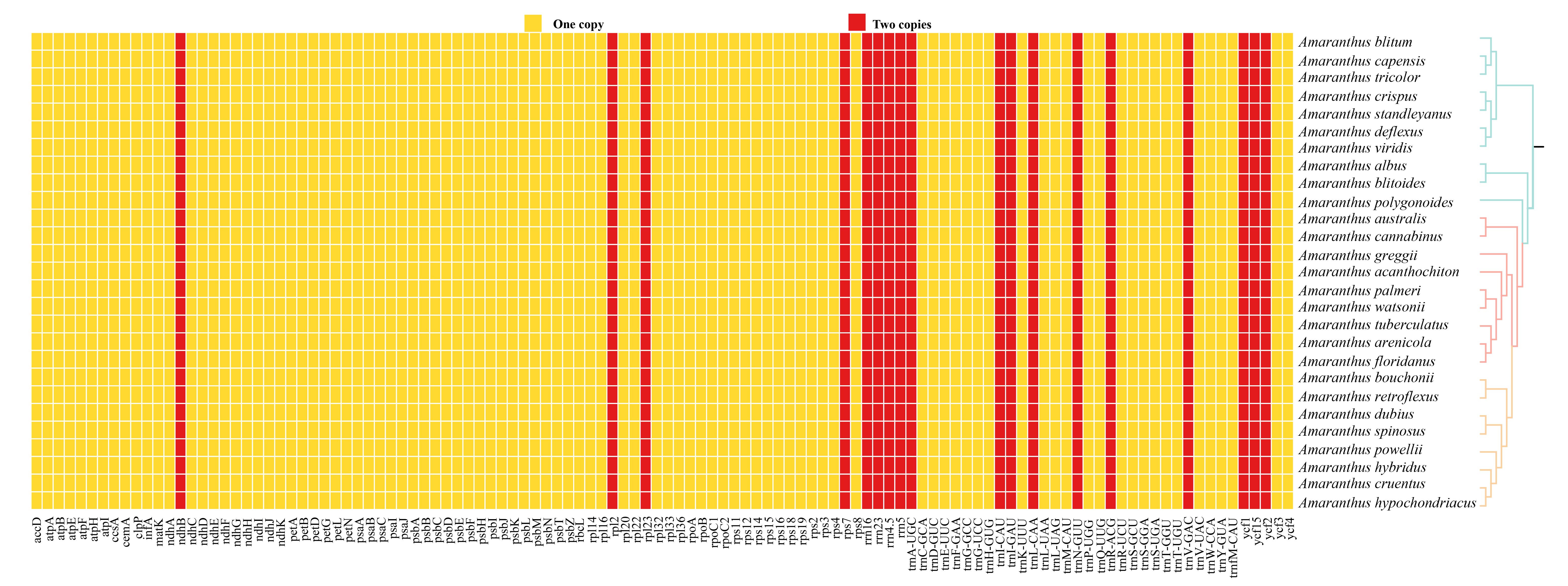

Supplement: Supplementary file 1 [file plants-14-00649-s001.zip › plants-3344668-supplementary/Figure S1.jpg]

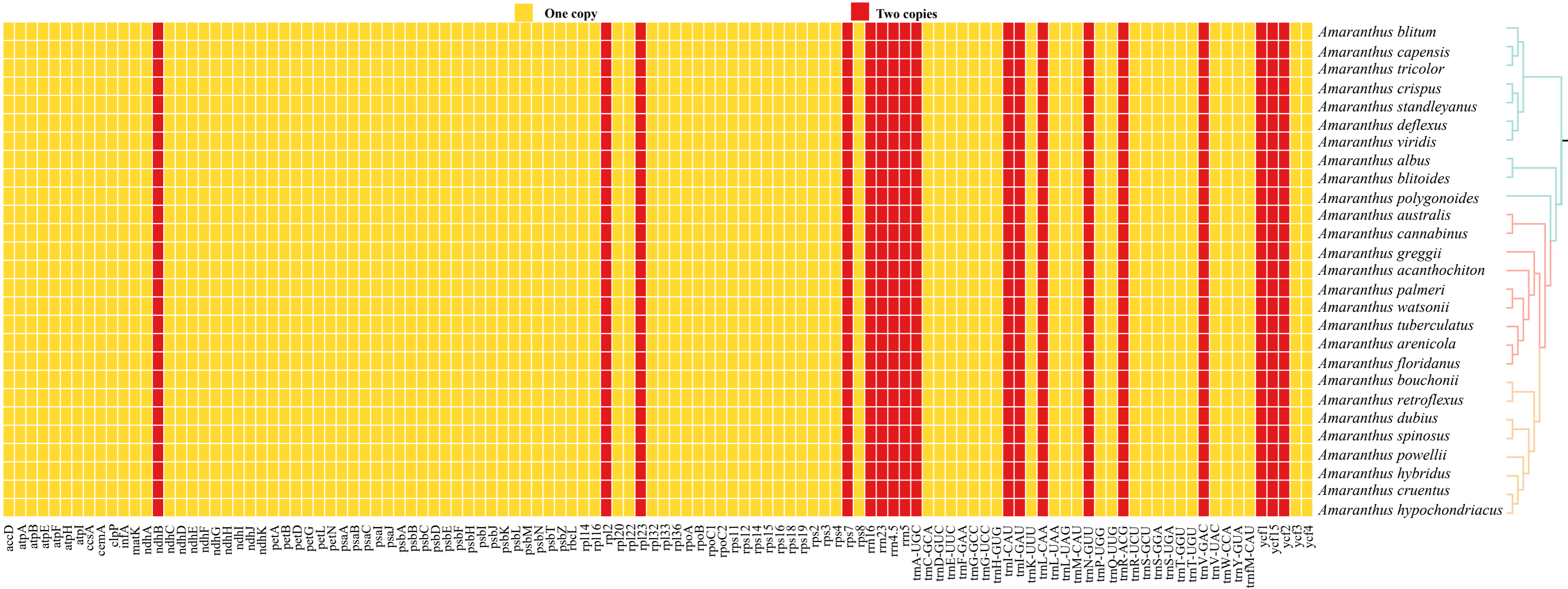

Supplement: Supplementary file 1 [file plants-14-00649-s001.zip › plants-3344668-supplementary/Figure S1.pdf]

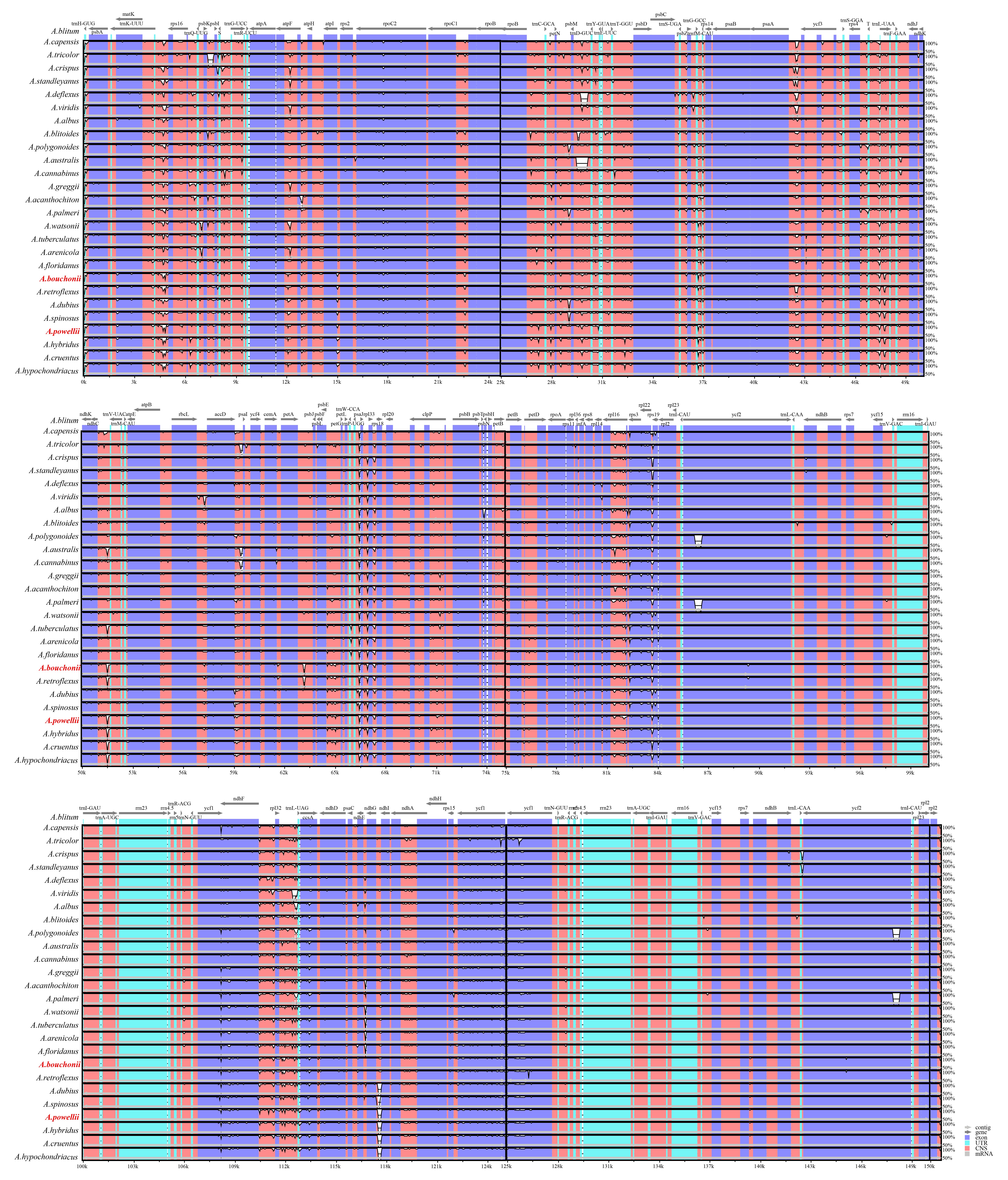

Supplement: Supplementary file 1 [file plants-14-00649-s001.zip › plants-3344668-supplementary/Figure S2.jpg]

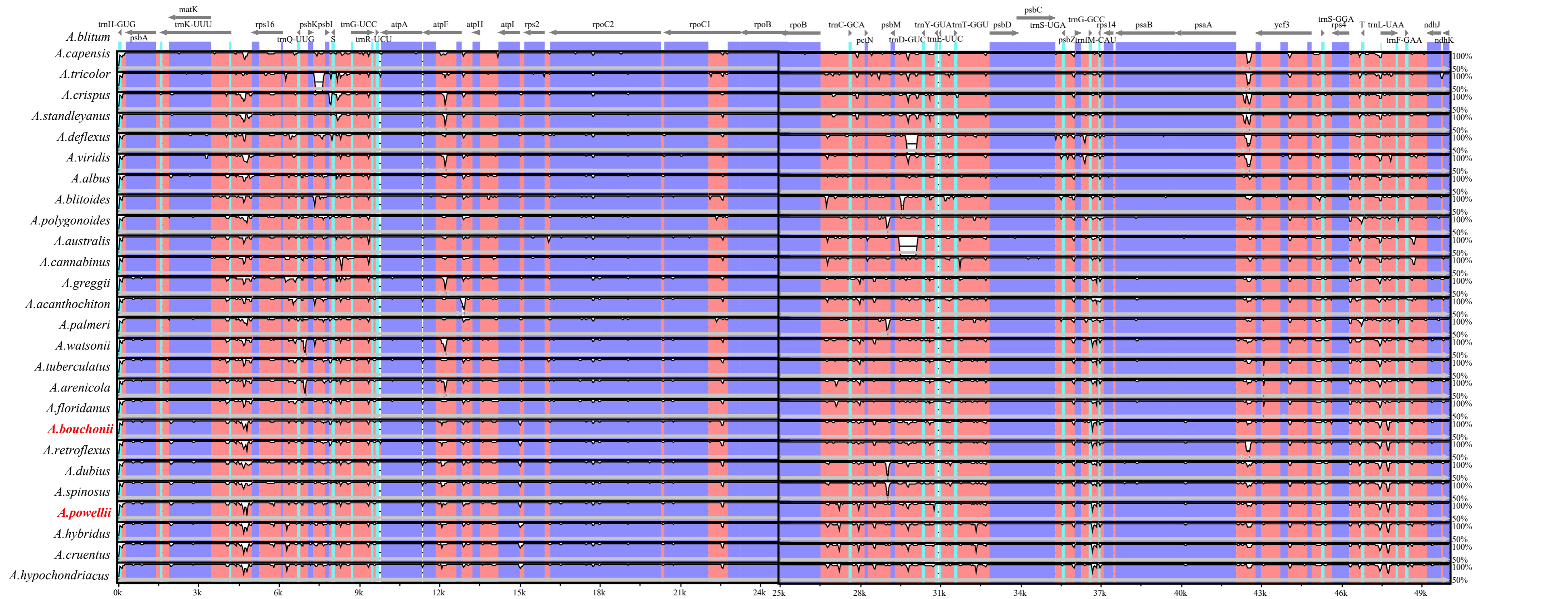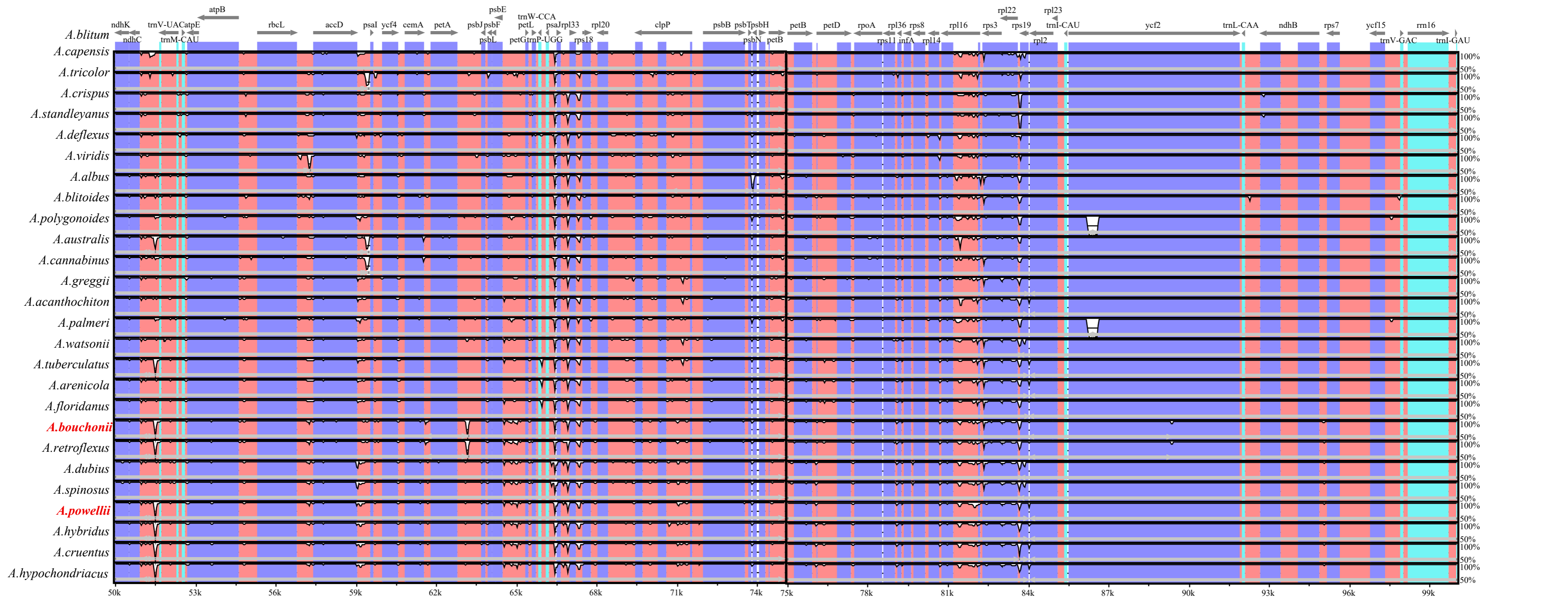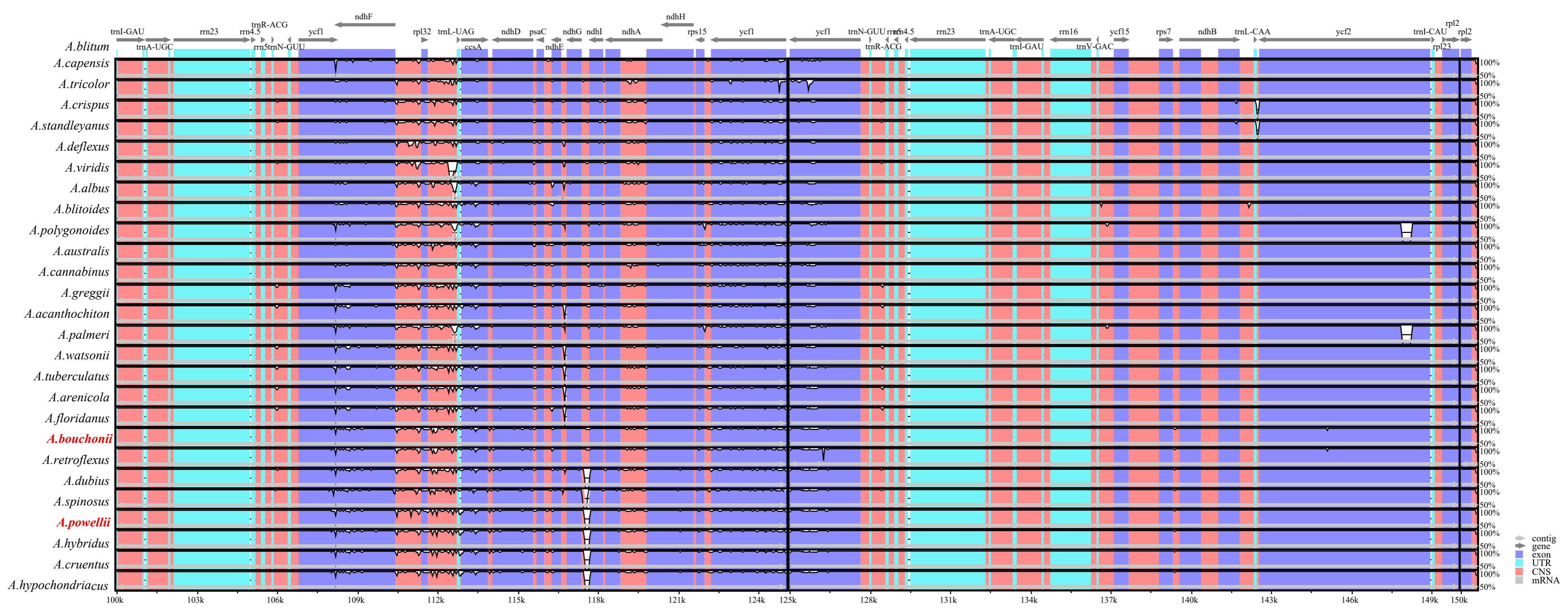

contig  
gene  
exon  
UTR  
CNS  
mRNA

Supplement: Supplementary file 1 [file plants-14-00649-s001.zip › plants-3344668-supplementary/Figure S2.pdf]

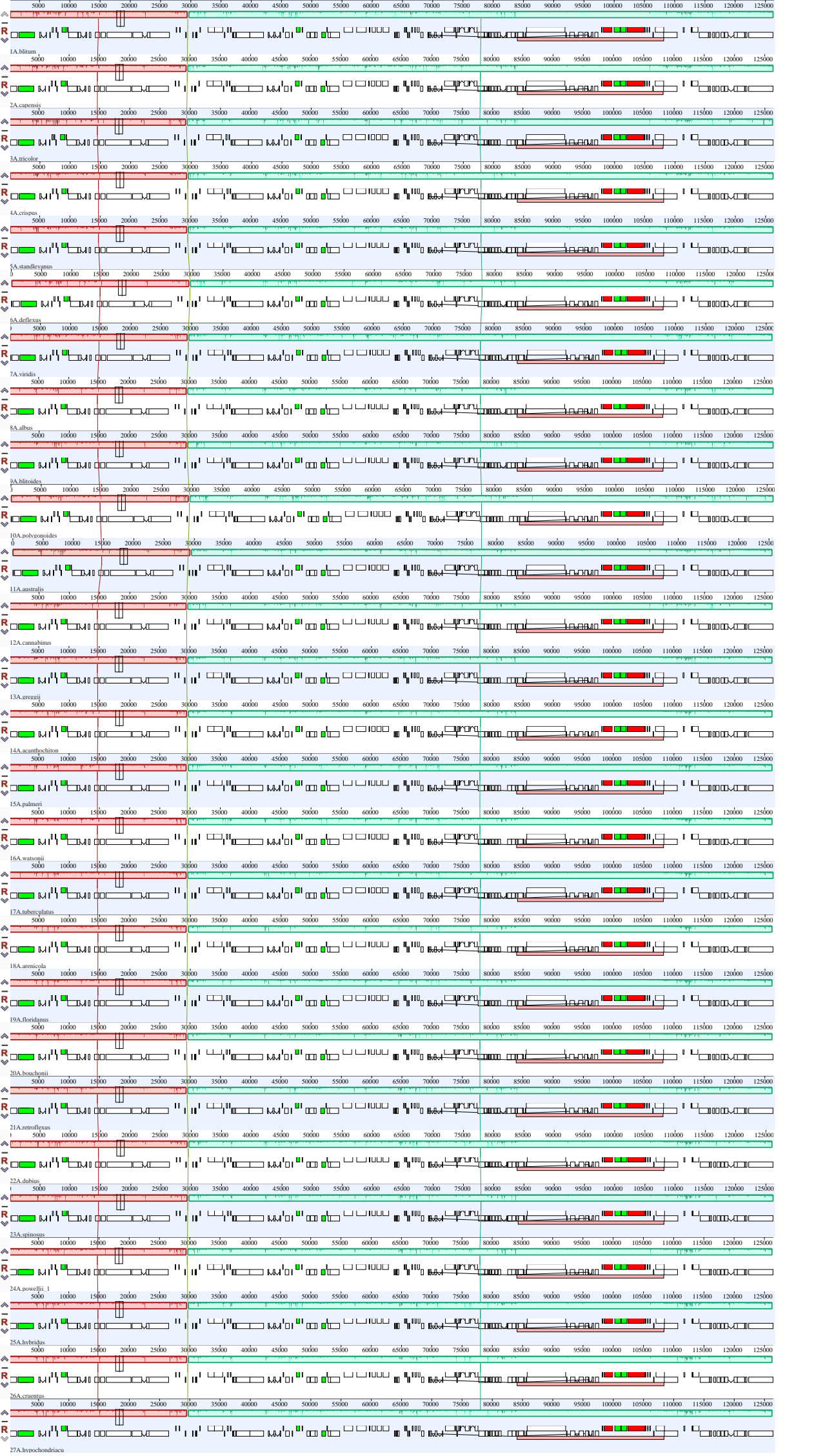

Supplement: Supplementary file 1 [file plants-14-00649-s001.zip › plants-3344668-supplementary/Figure S3.pdf]

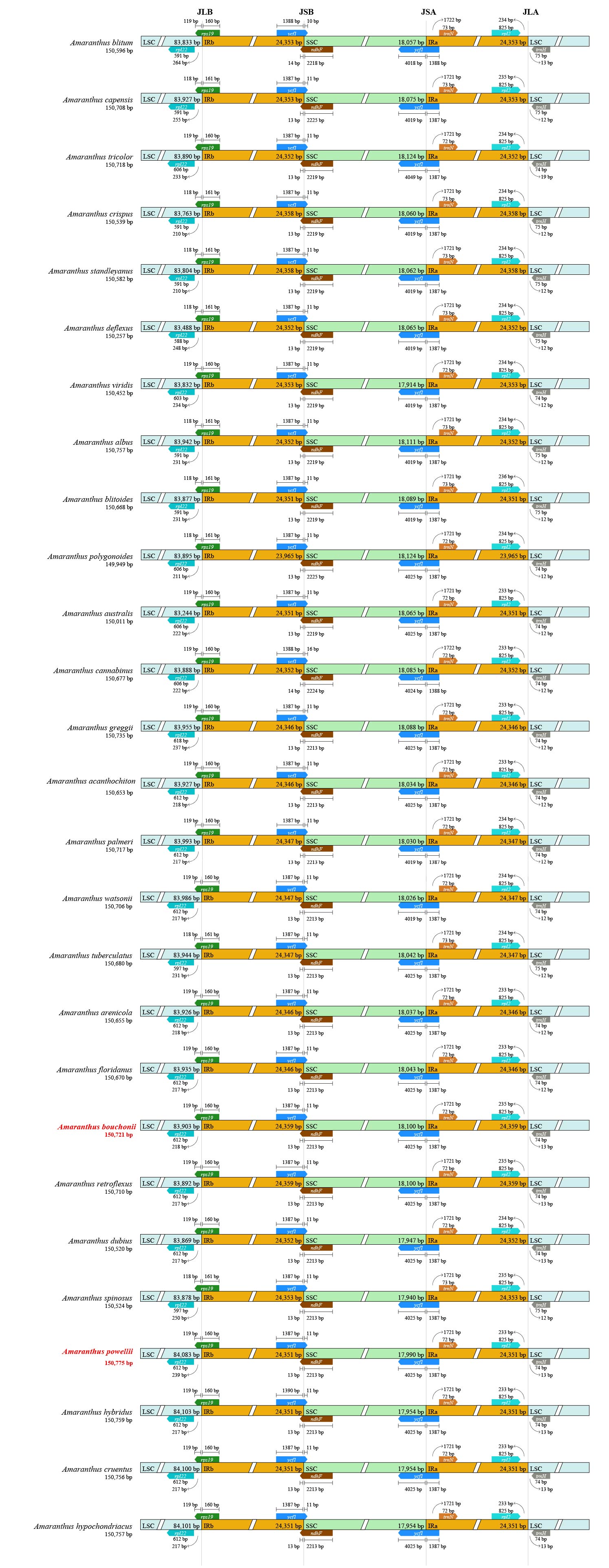

Supplement: Supplementary file 1 [file plants-14-00649-s001.zip › plants-3344668-supplementary/Figure S4 .jpg]

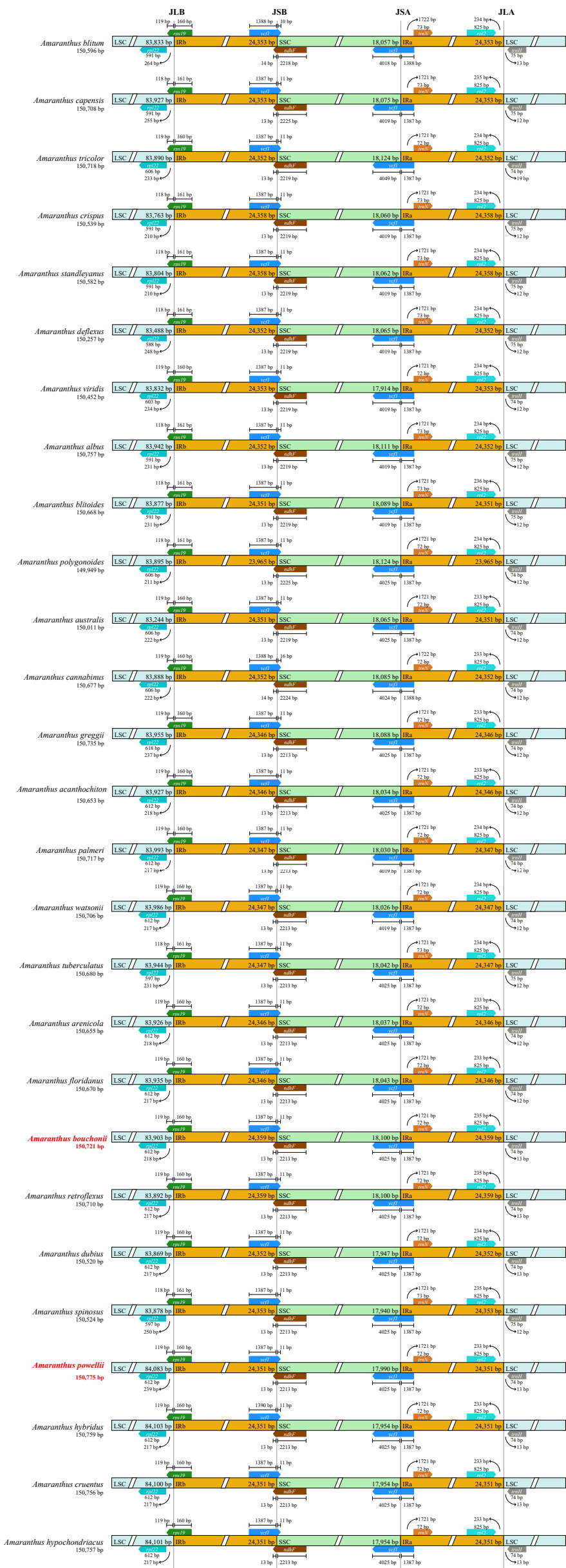

Supplement: Supplementary file 1 [file plants-14-00649-s001.zip › plants-3344668-supplementary/Figure S4 .pdf]
